# The Role of Phase Migration of Carbon Nanotubes in Melt-Mixed PVDF/PE Polymer Blends for High Conductivity and EMI Shielding Applications

**DOI:** 10.3390/molecules27030933

**Published:** 2022-01-29

**Authors:** Calin Lencar, Shashank Ramakrishnan, Elnaz Erfanian, Uttandaraman Sundararaj

**Affiliations:** Department of Chemical and Petroleum Engineering, University of Calgary, 2500 University Drive NW, Calgary, AB T2N 1N4, Canada; calin.lencar@ucalgary.ca (C.L.); shashank.ramakrishnan@ucalgary.ca (S.R.); elnaz.erfanian@ucalgary.ca (E.E.)

**Keywords:** polymer nanocomposite blends, phase migration, electrical conductivity, EMI shielding, poly(vinylidene fluoride) (PVDF), polyethylene (PE), multiwalled carbon nanotubes (MWCNTs)

## Abstract

In this work, the effects of blend ratio and mixing time on the migration of multi-walled carbon nanotubes (MWCNTs) within poly(vinylidene fluoride) (PVDF)/polyethylene (PE) blends are studied. A novel two-step mixing approach was used to pre-localize MWCNTs within the PE phase, and subsequently allow them to migrate into the thermodynamically favored PVDF phase. Light microscopy images confirm that MWCNTs migrate from PE to PVDF, and transmission electron microscopy (TEM) images show individual MWCNTs migrating fully into PVDF, while agglomerates remained trapped at the PVDF/PE interface. PVDF:PE 50:50 and 20:80 polymer blend nanocomposites with 2 vol% MWCNTs exhibit exceptional electromagnetic interference shielding effectiveness (EMI SE) at 10 min of mixing (13 and 16 dB, respectively-at a thickness of 0.45 mm), when compared to 30 s of mixing (11 and 12 dB, respectively), suggesting the formation of more interconnected MWCNT networks over time. TEM images show that these improved microstructures are concentrated on the PE side of the PVDF/PE interface. A modified version of the “Slim-Fast-Mechanism” is proposed to explain the migration behavior of MWCNTs within the PVDF/PE blend. In this theory, MWCNTs approaching perpendicular to the interface penetrate the PVDF/PE interface, while those approaching in parallel or as MWCNT agglomerates remain trapped. Trapped MWCNTs act as barriers to additional MWCNTs, regardless of geometry. This mechanism is verified via TEM and scanning electron microscopy and suggests the feasibility of localizing MWCNTs at the interface of PVDF/PE blends.

## 1. Introduction

Wireless electronic devices have become a staple in our everyday lives which has led to a surge in electromagnetic (EM) radiation being emitted into the environment. These EM waves can interfere with the effective operation of other adjacent devices, including navigational and medical equipment [1,2,3,4]. Traditionally, metals have been used to shield against electromagnetic interference (EMI), but they are expensive, heavy, difficult to process, and susceptible to corrosion [5,6]. Furthermore, metals attenuate incident EMI by reflecting it back into the environment [7,8]. Recent works have sought to remedy the limitations of metals via the creation of metal-based polymer nanocomposites [9,10,11]. Ji et al. in particular [9] made use of Cu-Ni-carbon nanotube (CNT) open-celled foams, which relied on multiple reflections to attenuate incident EMI. Ultimately, these methods are limited by the need to use high concentrations of nanofiller and require expensive solution-based methods to synthesize. Polymer nanocomposites (PNCs) with multiwalled carbon nanotubes (MWCNTs) have attracted great interest as conductive materials that can be used as EMI shields that attenuate incident EMI by absorbing it, reducing the amount of EM smog in the environment [12,13,14]. MWCNTs possess exceptional conductivity, tensile strength, and a high aspect ratio (AR), which allows them to effectively impart their electrical properties within a polymer matrix when embedded therein, by forming interconnected conductive networks [12,15,16,17]. More recent works have even made use of hybrid CNT fillers to achieve exceptional charge storage, and flame retardant properties [18,19]. Samy et al. [19] used tetrabenzonaphthalene-based conjugated microporous polymers coated onto single-walled carbon nanotubes to make a nanocomposite material with exceptional capacitance and thermal stability. The main limitation facing the widespread application of MWCNT-based PNCs is the high cost of synthesizing MWCNTs. Due to this prohibitively high cost, researchers have been investigating ways of reducing the amount of MWCNTs needed to create low-cost and high conductive PNCs. 

Recent works have shown that immiscible polymer blend nanocomposites (PBNs) containing MWCNTs offer great potential for the development of high performance, low-cost conductive materials. Sumita et al. [20,21] initially demonstrated that by localizing carbon black (CB) within the matrix phase of a PBN, you could effectively alter its local concentration, thereby lowering the amount of CB needed to form a percolated network within the system. In these systems, it is vital that the morphology of the binary polymer mixture is co-continuous, such that the percolation of nanofiller within one of the phases results in a continuous conductive network structure. This phenomenon is known as double percolation and has since been applied extensively to numerous PBN systems containing MWCNTs [12,15,16]. The percolation threshold for MWCNTs within polymer blends can be reduced even further by localizing them at the interface of the blend. Since the relative volume occupied by the interface of an immiscible blend system is extremely low, interfacial localization of MWCNT would allow for extremely low percolation thresholds to be achieved [22,23]. Previous works have shown percolation threshold concentrations as low as 0.025 vol% using MWCNTs [24,25]. The main drawback of interfacial localization is that functionalized MWCNTs are often used which are significantly more costly to synthesize, and often have compromised electrical properties, or additional additives must be used, which in turn drive up the manufacturing cost. 

Predicting the movement of MWCNTs within a given polymer blend is crucial to achieving interfacial localization. MWCNTs must initially be localized in the phase with lower thermodynamic affinity, to encourage their migration toward their preferred phase, and by extension, the interface of the blend system. A common method for predicting the thermodynamic preference of MWCNTs within a polymer blend is a modified Young’s equation, adapted by Sumita et al. [20]:

(1) $$\omega_{A\;\mathrm{to}\;B}=\frac{\sigma_{A/\mathrm{MWCNT}}-\sigma_{\mathrm{MWCNT}/B}}{\sigma_{A/B}}$$

where $\omega_{A\;\mathrm{to}\;B}$ represents the wettability of MWCNTs within a binary blend of polymer A and B, $\sigma_{A/B}$ is the surface energy between polymer A and B, $\sigma_{A/\mathrm{MWCNT}}$ is the surface energy between the nanofiller and polymer A, and $\sigma_{\mathrm{MWCNT}/B}$ is the surface energy between the nanofiller and polymer B. If $\omega_{A\;\mathrm{to}\;B}>1$, MWCNTs will favor polymer A; if $\omega_{A\;\mathrm{to}\;B}<-1$, MWCNTs will prefer polymer B; if $-1<\omega_{A\;\mathrm{to}\;B}<1$, MWCNTs will tend to remain at the interface of polymer A and B [20].

Because MWCNTs must be pre-localized in the thermodynamically unfavorable phase, the order of mixing for the polymer system is crucial to achieving interfacial localization. This can be accomplished by either premixing MWCNTs in the thermodynamically unfavorable phase via a masterbatch, or by making sure that the unfavorable phase has a lower melting temperature [4]. Several works have shown that the viscosity of the pre-localized phase, as well as the viscosity of the destination phase, have a large impact on the final localization of nanofiller [23,26,27,28]. Sun et al. used polystyrene (PS)/acrylonitrile butadiene styrene (ABS) blends containing MWCNTs at varying viscosities of ABS and found that MWCNTs could migrate more easily toward the thermodynamically favored PS phase if the pre-localized ABS phase had a low viscosity, and they were more likely to become trapped in ABS if it had a higher viscosity [29]. Similar behavior was observed in systems of polyamide 12 (PA12)/polyethylene (PE)/MWCNTs [30] and polylactic acid (PLA)/polycaprolactone (PCL)/MWCNTs [31], resulting from the unfavorably high viscosity of the pre-localized phase with lower thermodynamic affinity for MWCNTs. It is therefore vital that the viscosity of the pre-localized phase should be kept low, while the viscosity of the destination phase should be kept high, if interfacial localization of MWCNTs is desired. 

Other factors that can impact the viscosity of the polymer phases include the processing temperature and the mixing speed of the mixing apparatus, both of which reduce the viscosity of the polymer as they are increased. A reduction in the viscosity of the pre-localized phase permits easier travel of MWCNTs toward the interface of the system [32]. Lower viscosity of the destination phase allows MWCNTs to more easily enter once they penetrate the interface. Furthermore, as the mixing speed is increased, the shear forces applied on the blend system result in the more rapid formation of new interface as the morphology is kneaded and broken up. This creation of new interface increases the likelihood of MWCNTs encountering the polymer/polymer interface as they migrate, which in turn can result in either interfacial entrapment, or complete migration into the destination phase [33]. Increased shear forces also lead to more scission of nanotubes. Shorter MWCNTs are less likely to coil or entangle with one another and are consequently more effective at penetrating the polymer/polymer interface [34].

The main objective of this work was to understand the role that the polymer blend ratio and mixing time plays on the phase migration of MWCNTs within an immiscible polymer blend of poly(vinylidene difluoride) (PVDF) and polyethylene (PE), and to subsequently use this understanding to develop a PBN material with high conductivity, EMI shielding effectiveness and high rheological properties. MWCNTs were the nanofiller of choice, owing to their exceptional electrical and mechanical properties, their high AR, and their low cost to manufacture, compared to similar nanoparticles such as SWCNTs [15]. Furthermore, MWCNTs boast greater corrosion and oxidation resistance compared to SWCNTs, since only the outermost nanotube layer is exposed to chemical attack. PVDF was chosen for its high toughness, the desirable piezoelectric properties of its crystalline β-phase, and high thermodynamic affinity for MWCNTs [35]. PE was chosen for its low cost, easy processability, and its low thermodynamically affinity for MWCNTs [36]. It was found that the blend ratio of PVDF:PE had a significant impact on the speed and extent of MWCNT migration by the 10 min mark. A theoretical thermodynamic approach using a modified Young’s equation predicted the polymer phase in which the MWCNTs would have to lowest interfacial energy. It was found that MWCNTs preferred the PVDF phase and were thus pre-localized in the PE phase to observe migration. Light microscopy (LM) and transmission electron microscopy (TEM) were both used to confirm the validity of the theoretical model used. The observed network structures of MWCNTs within the prepared PBN samples were correlated with the electrical and rheological properties.

## 2. Materials and Methods

### 2.1. Materials and Sample Preparation

PBN samples were prepared using PVDF (Dyneon^TM^ 11008/0001) supplied by the 3M Advanced Materials Division (St. Paul, MN, USA), PE (Lumicene^TM^ M3581uv) supplied by Total, and MWCNTs (NC7000) supplied by Nanocyl S.A. (Sambreville, Belgium). According to supplier specifications, NC 7000 had an average diameter of 9.5 nm, an average length of 1.5 µm, 90% purity and an electrical conductivity of 10^6^ S/m. PVDF and PE were vacuum dried at 60 °C for 24 h prior to use. 

PBN samples of three different blend ratios (PVDF:PE 80:20, 50:50 and 20:80) with an overall MWCNT concentration of 2 vol% were prepared via a multistep melt mixing process. The initial PE/MWCNT masterbatch (MB) was prepared at a concentration of 20 wt% MWCNTs in a Process 11 twin-screw mini-extruder (Thermofisher Scientific, Waltham, MA, USA) with a temperature profile ranging from 120–200 °C and a rotor speed of 50 rpm. The produced PE/MWCNT MB was combined with additional pure PE powder in the mixing cup of an Alberta Polymer Asymmetric Mini-mixer (APAM) (University of Calgary, Calgary, AB, Canada) and allowed to melt for 1 min with no rotation [37]. The mixture was then blended at 200 rpm for 3 min, to create a homogeneous PE phase containing MWCNTs. Mixing was then stopped, and PVDF powder was added into the mixing cup, and allowed to melt for 3 min with no rotation. Finally, the combined mixture was mixed at 200 rpm for 0.5, 1, 5, and 10 additional min. All melting and mixing steps within the APAM were performed at a constant temperature of 200 °C. Small chunks of sample were rapidly removed at the end of mixing and quenched in liquid nitrogen to freeze the melt morphology, while most of the sample material was taken and compression molded using a Carver compression molder (model 3912) (Carver Inc., Wabash, IN, USA) into circular discs (diameter = 25 mm, thickness = 0.45 mm) at 200 °C and 35 MPa for 10 min. A least 4 specimens were prepared for electrical conductivity, EMI shielding and rheological measurements. Each specimen was marked, such that its electrical and rheological properties could be directly compared.

### 2.2. Sample Characterization

For LM imaging, thin sections of roughly 2 µm in thickness were prepared using a Leica EM UC6 ultramicrotome (Leica Microsystems, Wetzlar, Germany) at −140 °C, and imaged using an Olympus^®^ BX60 optical microscope (Olympus Inc, Tokyo, Japan) connected to an Olympus DP80 camera. TEM imaging was also performed by first making 80 nm sections using a Leica Ultracut UTC ultramicrotome, and then taking TEM micrographs with a Hitachi H-7500 TEM (Hitachi High-Tech Corp., Tokyo, Japan) equipped with an Olympus SIS MegaView II 1.35 MB digital camera. Preliminary backscattered electron (BSE) scanning electron microscope (SEM) images were taken using a Thermofisher Scientific Phenom ProX Desktop SEM (Thermofisher Scientific, Waltham, MA, USA) to ascertain the bulk morphologies of the PVDF and PE phases. For detailed SEM imaging, cryo-fractured samples were mounted and imaged under a low vacuum using a Quanta FEG 250 VP-FESEM (variable pressure field emission SEM) (FEI Company, Hillsboro, OR, USA). Back-scattered electron images were taken to differentiate between the PVDF and PE phases. A large field detector (LFD) was used to take secondary electron images to observe the sample topography at a nanoscale.

**Figure 1:**
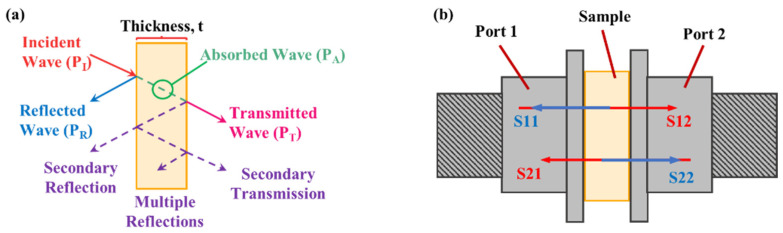
Diagrams of (**a**) the three EMI shielding mechanisms and (**b**) the scattering parameters studied in this work. For details on the scattering parameters, refer to the Supplementary Materials.

The EMI shielding effectiveness (EMI SE) of a material represents its ability to block incident EMI, which can be achieved through three mechanisms: absorption (EMI SE_A_), reflection (EMI SE_R_), and multiple reflections (EMI SE_MR_). A depiction of the three shielding mechanisms can be seen in Figure 1a. If the thickness of the shield is greater than its skin depth, the contribution of multiple reflections can be neglected. Consequently, the total shielding effectiveness (EMI SE_T_) can be expressed as ${\mathrm{EMI}\;\mathrm{SE}}_{T}={\mathrm{EMI}\;\mathrm{SE}}_{R}+{\mathrm{EMI}\;\mathrm{SE}}_{A}$ [38]. EMI measurements were performed in the X-band frequency range (8.2–12.4 GHz) using a vector network analyzer (ENA Model E5071C) (Agilent Technologies, Santa Clara, CA, USA) connected to a WR-90 rectangular waveguide. EMI SE values were calculated using scattering parameters (Figure 1b) derived from the measured data [39].

DC electrical conductivity of the compression molded samples was measured using a Loresta GP (model MCP-T610) resistivity meter (Mitsubishi Chemical Co., Tokyo, Japan), attached to a four-pin ESP probe. Measurements were performed on four specimens for each sample, and the average conductivity and EMI SE values were reported with error bars (one standard deviation).

All rheological tests were performed using an Anton-Paar MCR 302 rheometer (Anton Paar GmbH, Graz, Austria) equipped with a 25 mm diameter parallel plate and gap size of 0.40–0.50 mm. Frequency sweeps were performed within the linear viscoelastic (LVE) region at a temperature of 200 °C constant strain of 0.5%, to study any potential MWCNT microstructures contained within the PBN samples without destroying them. Frequency sweeps on each sample were followed-up with strain sweeps, to confirm that the frequency sweeps were performed within the LVE region, and to further study the microstructure within the samples. 

## 3. Results

### 3.1. Predicting Thermodynamic Affinity

A modified Young’s equation was used to predict which phase MWCNTs would thermodynamically favor. The interfacial surface energy between each phase was calculated using both the harmonic and geometric means [40] of the surface free energies of the individual components, which were obtained from literature [40,41]. The results of the wettability calculations are summarized in Table 1. For more details on the surface energies taken from literature, calculations of additional surface energy data, and the use of the harmonic and geometric means, please refer to the Supplementary Materials.

Based on the values calculated above, MWCNTs strongly prefer PVDF over PE, to the point where they will fully migrate into PVDF. Thus, MWCNTs should be pre-localized in the PE phase, and driven toward PVDF through subsequent melt-mixing.

### 3.2. Imaging Results

**Figure 2:**
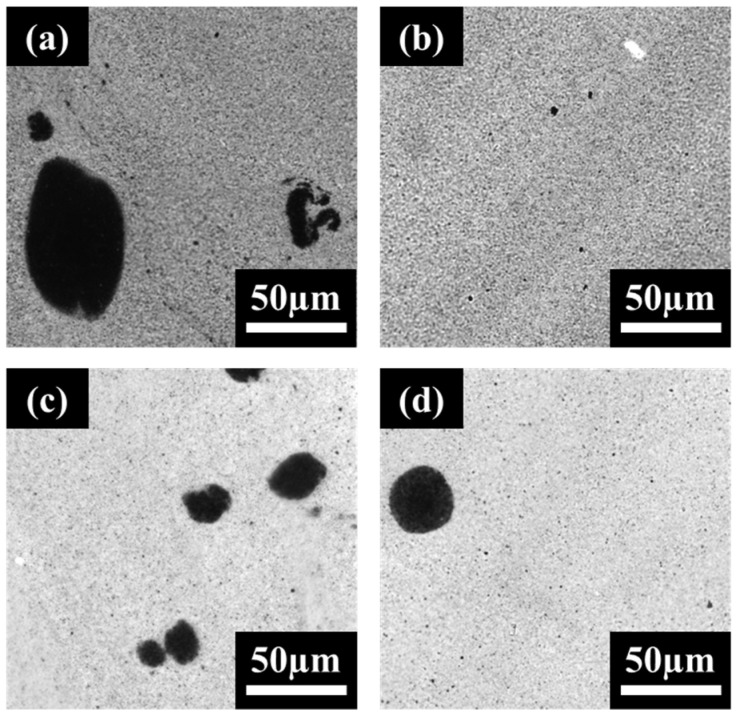
LM images for neat PE/MWCNT 2 vol% mixed for (**a**) 1 min and (**b**) 10 min, and PVDF/MWCNT 2 vol% mixed for (**c**) 1 min and (**d**) 10 min.

LM imaging was first performed on PVDF and PE nanocomposites, to identify how MWCNTs disperse within each polymer. Figure 2 below shows the dispersion of MWCNTs within PVDF and PE after 1 and 10 min of mixing. The PE samples (Figure 2a) contain significantly larger agglomerates than PVDF (Figure 2c) at the 1 min mark; however by 10 min, the dispersion of MWCNTs within PE (Figure 2b) is only slightly less fine than within PVDF (Figure 2d). By 10 min, both PVDF and PE show a high-quality dispersion of MWCNTs, which suggests that the proposed two-step mixing method is highly effective at creating a homogeneous MWCNT-rich phase.

**Figure 3:**
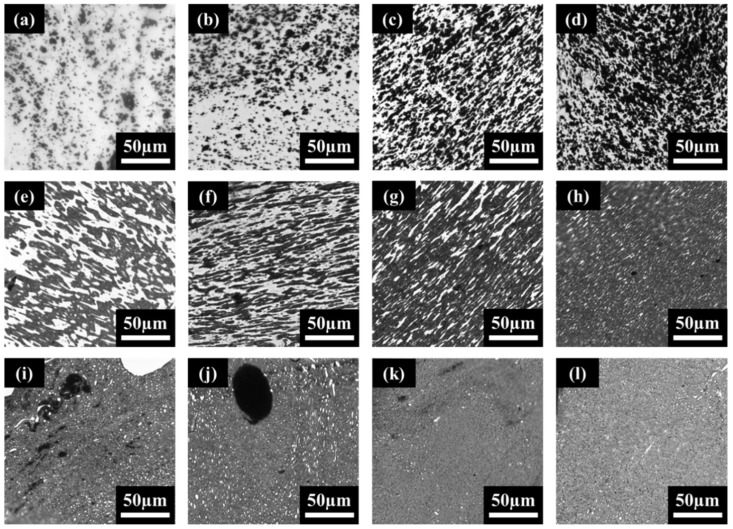
LM images for PVDF:PE blends with 2 vol% MWCNTs, including—80:20 blends mixed for (**a**) 0.5 min, (**b**) 1 min, (**c**) 5 min and (**d**) 10 min; 50:50 blends mixed for (**e**) 0.5 min, (**f**) 1 min, (**g**) 5 min and (**h**) 10 min; 20:80 blends mixed for (**i**) 0.5 min, (**j**) 1 min, (**k**) 5 min and (**l**) 10 min.

Next, LM images were taken of the prepared PVDF:PE samples (Figure 3). The dark phases seen in Figure 3a,e,i (all at 30 s of mixing time) are MWCNT-rich PE, while the light phase is PVDF. As the mixing time is increased for all compositions, the surface area of the LM cross-section occupied by the dark phase increases. Since the blend composition remains constant during mixing, the increase in the surface area occupied by the dark phase is likely caused by a migration of MWCNTs toward the PVDF/PE interface, and into the PVDF phase. Additionally, as the volume fraction occupied by PE is increased, the hue of the dark phase observed becomes increasingly light. This is due to the homogeneous MWCNT-rich PE phase containing a lower concentration of MWCNTs as the volume fraction of PE is increased (overall MWCNT volume fraction is kept constant for all samples). Notably, the MWCNT-rich PE droplets seen in the PVDF:PE 80:20 samples (Figure 3a–d) are dark, which suggests that each droplet is filled with MWCNT agglomerates. In contrast, the MWCNT-filled PE phase seen in the 20:80 samples (Figure 3i–l) is far lighter, and individual, dark MWCNT agglomerates can be observed. 

**Figure 4:**
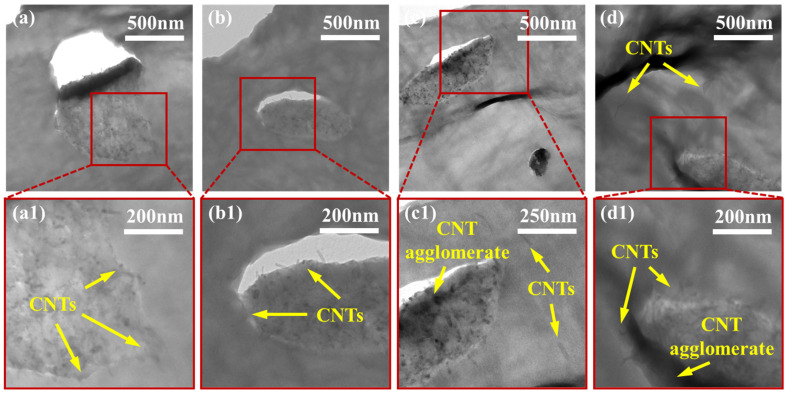
TEM images for PVDF:PE 80:20 blends with 2 vol% MWCNTs mixed for (**a**,**a1**) 0.5 min, (**b**,**b1**) 1 min, (**c**,**c1**) 5 min and (**d**,**d1**) 10 min.

**Figure 5:**
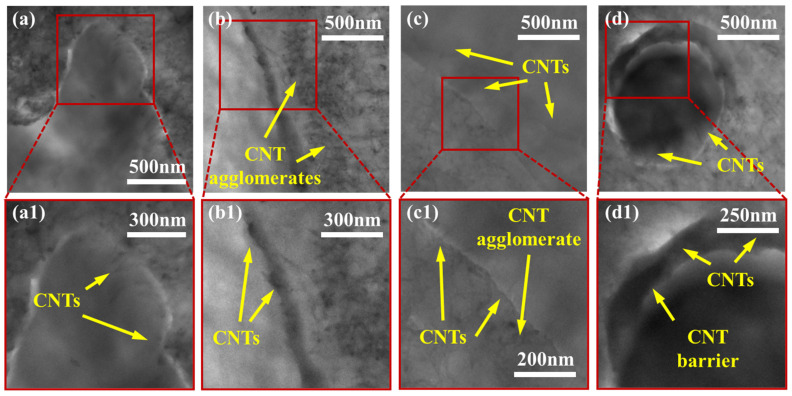
TEM images for PVDF:PE 50:50 blends with 2 vol% MWCNTs mixed for (**a**,**a1**) 0.5 min, (**b**,**b1**) 1 min, (**c**,**c1**) 5 min and (**d**,**d1**) 10 min.

**Figure 6:**
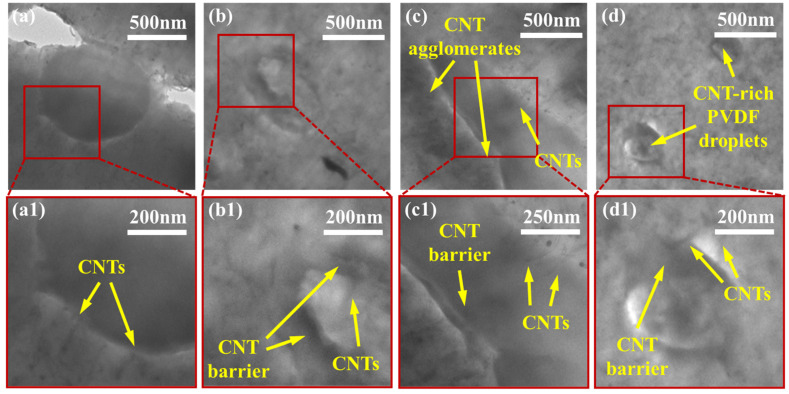
TEM images for PVDF:PE 20:80 blends with 2 vol% MWCNTs mixed for (**a**,**a1**) 0.5 min, (**b**,**b1**) 1 min, (**c**,**c1**) 5 min and (**d**,**d1**) 10 min.

To effectively study the exact localization of smaller MWCNT agglomerates and individual MWCNTs, TEM imaging was used. Figure 4, Figure 5 and Figure 6 show the localization of MWCNTs within the PVDF:PE 80:20, 50:50 and 20:80 blends, respectively. All PVDF:PE samples show that MWCNTs are predominantly localized within the PE phase. Figure 4 shows the changes in the localization of MWCNTs for PVDF:PE 80:20 samples as the mixing time is increased. At 30 s (Figure 4a,a1), a PE droplet within the PVDF matrix loaded with a dense network of MWCNTs appears almost as a single agglomerate. Closer inspection of the PVDF/PE interface shows that some MWCNTs within the PE phase are already beginning to penetrate the PVDF phase. As the mixing time is increased. The quantity of MWCNTs straddling the PVDF/PE interface continues to increase. At 5 and 10 min of mixing time (Figure 4c,c1,d,d1), individual MWCNTs that have completely migrated into the PVDF phase can be observed. 

Figure 5 shows the localization of MWCNTs within the PVDF:PE 50:50 samples with increasing mixing time. For the 50:50 samples, many MWCNTs can be seen penetrating into PVDF. This is a consequence of the higher interfacial surface area between PVDF and PE in these samples, which increases the likelihood of individual MWCNTs and MWCNT agglomerates encountering the PVDF:PE interface during mixing. At 30 s of mixing (Figure 5a,a1), numerous MWCNTs can be seen penetrating the PVDF/PE interface. At 1 min of mixing (Figure 5b,b1), individual MWCNTs localized completely within PVDF can be seen. This trend continues for 5 and 10 min of mixing, with even more individual MWCNTs present within PVDF. As the mixing time is increased, the position of MWCNT agglomerates within the PE phase changes. At the onset of mixing, MWCNT agglomerates appear to be dispersed uniformly throughout the PE phase. However, as the mixing time is increased, MWCNT agglomerates begin to migrate toward the PVDF/PE interface, and individual MWCNTs within the agglomerates poke through into the PVDF phase. By 10 min of mixing, smaller PVDF droplets become surrounded with dark rings that are filled with MWCNTs. These rings are likely PE richly saturated with MWCNT agglomerates, that have migrated toward PVDF, but are unable to penetrate the interface.

Figure 6 shows the localization of MWCNTs within PVDF:PE 20:80 samples as mixing time is increased. At 30 s of mixing (Figure 6a,a1), there is little penetration of MWCNTs from the PE phase into the PVDF phase. The lower concentration of MWCNTs within the more voluminous PE phase, coupled with the lower interfacial surface area existing between the PVDF droplets and the PE matrix results in a reduced likelihood of MWCNTs encroaching on the interface at the onset of mixing. At 1 min of mixing (Figure 6b,b1), smaller PVDF droplets begin to be invaded by MWCNTs penetrating through the PVDF/PE interface. At 5 min of mixing (Figure 6c,c1), MWCNTs can be seen straddling the PVDF/PE interface, and small agglomerates of MWCNTs can be seen concentrating into dark domains that surround the PVDF droplets and fibers. At 10 min of mixing (Figure 6d,d1) smaller PVDF droplets have become saturated with MWCNTs, and are more difficult to distinguish from the PVDF phase. The reduced concentration of MWCNTs within PE in the 20:80 samples result in fewer MWCNT agglomerates being present within the blend.

**Figure 7:**
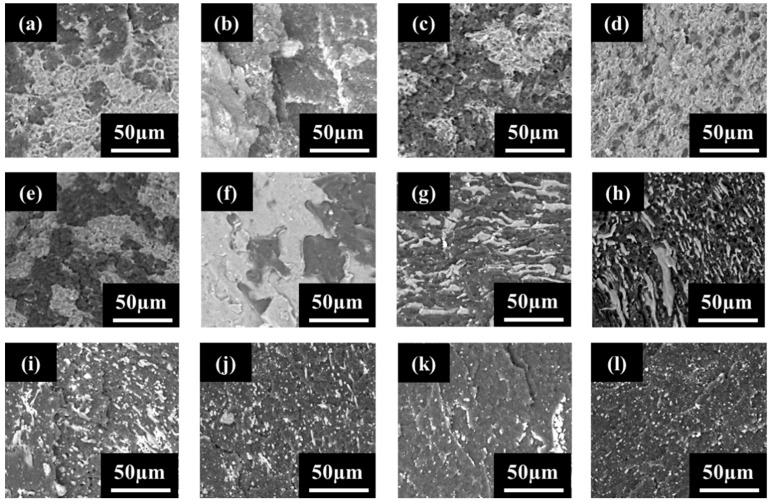
Preliminary BSE SEM images for PVDF:PE blends with 2 vol% MWCNTs, including—80:20 blends mixed for (**a**) 0.5 min, (**b**) 1 min, (**c**) 5 min and (**d**) 10 min; 50:50 blends mixed for (**e**) 0.5 min, (**f**) 1 min, (**g**) 5 min and (**h**) 10 min; 20:80 blends mixed for (**i**) 0.5 min, (**j**) 1 min, (**k**) 5 min and (**l**) 10 min.

Figure 7 shows BSE images of PVDF:PE 80:20 blends (Figure 7a–d), 50:50 blends (Figure 7e–h), and 20:80 blends (Figure 7i–l) mixed for 30 s, 1 min, 5 min, and 10 min. The lighter phase in all samples is PVDF, whilst the darker phase is the MWCNT-rich PE. As seen in the LM images, as the mixing time is increased, the minor phase within the sample becomes increasingly dispersed, due to shear forces from the matrix phase having more time to breakup the minor phase. For the 80:20 samples, by 10 min of mixing, the PE phase has become finely dispersed, that it is nearly impossible to differentiate from PVDF, even with high contrast BSE images. In the case of the 50:50 samples, the initial morphology of the blend appears co-continuous (e.g., 30 s and 1 min of mixing), but the PVDF phase becomes dispersed by 10 min of mixing. 

**Figure 8:**
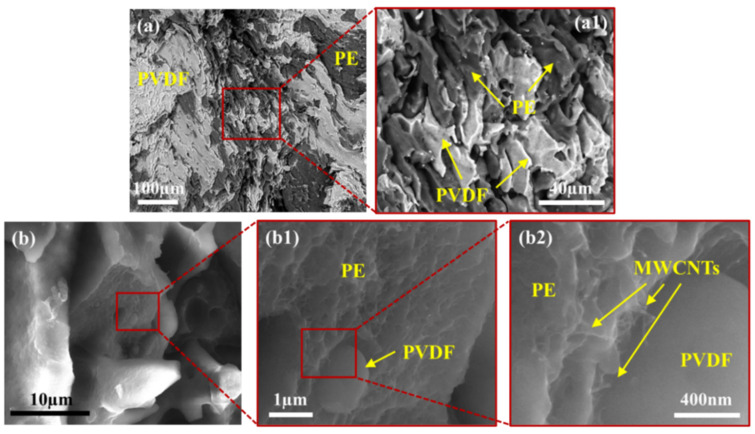
SEM images of PVDF:PE 50:50 with 2 vol% MWCNTs mixed for 1 min. (**a**,**a1**) BSE and (**b**,**b1**,**b2**) LFD images are included.

**Figure 9:**
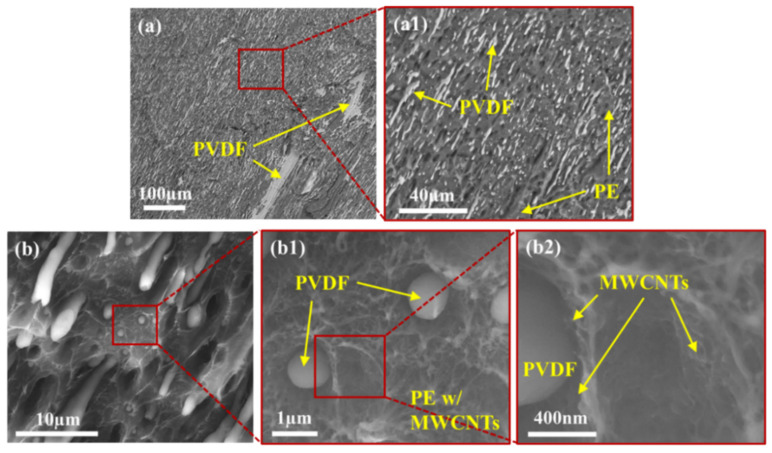
SEM images of PVDF:PE 50:50 with 2 vol% MWCNTs mixed for 10 min. (**a**,**a1**) BSE and (**b**,**b1**,**b2**) LFD are included.

Figure 8 shows detailed BSE and LFD images for the PVDF:PE 50:50 sample mixed for 1 min. The BSE images (Figure 8a) show a coarse, co-continuous morphology of PVDF and PE, which matches closely with LM images for the same sample (Figure 3e). Due to the short mixing time, the morphology is coarse, with sheet-like structures of PVDF and PE being observed (Figure 8a1). LFD images (Figure 8b) show the fibrous structure of the PE phase, which indicates the presence of a MWCNT network structure. Conversely, the PVDF phase is smoother, which indicates an absence of MWCNTs. Small ridges surrounding the PVDF phase can be seen, which are likely due to an increased concentration of MWCNTs along the PVDF/PE interface. Figure 9 shows the morphology of PVDF:PE 50:50 at 10 min of mixing. BSE images (Figure 9a) show a far finer morphology, with PE appearing continuous, whereas PVDF has become dispersed. The breakup of the PVDF phase is due to its lower viscosity compared to PE (refer to Supplementary Materials for rheological data of the pure polymers), which has a higher viscosity because it is loaded with MWCNTs. LFD images (Figure 9b) show more pronounced ridges around the PVDF phase along the fracture surface, likely caused by the presence of MWCNTs in PE.

**Figure 10:**
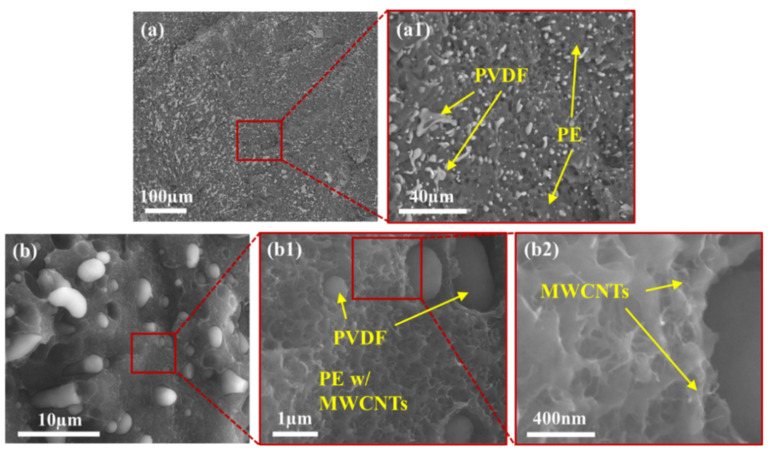
SEM images of PVDF:PE 20:80 with 2 vol% MWCNTs mixed for 1 min. (**a**,**a1**) BSE and (**b**,**b1**,**b2**) LFD images are included.

**Figure 11:**
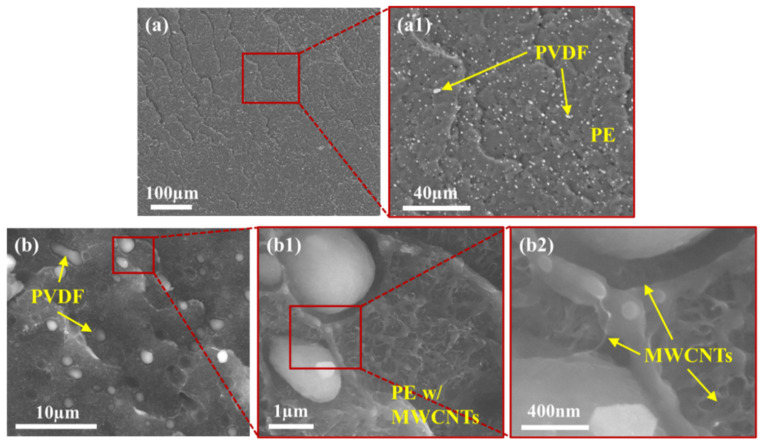
SEM images of PVDF:PE 20:80 with 2 vol% MWCNTs mixed for 10 min. (**a**,**a1**) BSE and (**b**,**b1**,**b2**) LFD images are included.

Figure 10 shows the BSE and LFD images for the PVDF:PE 20:80 sample mixed for 1 min. BSE images (Figure 10a) show a relatively fine dispersion of PVDF droplets, within a continuous PE phase. As with the 50:50 1 min sample, LFD images of PVDF:PE 20:80 show that MWCNTs lie within the rough PE phase, and the PVDF phase appears very smooth comparatively. Furthermore, PE ridges can be seen again surrounding the PVDF droplets. As the mixing time is increased to 10 min, the dispersion of PVDF droplets becomes much finer, as seen in Figure 11a. As with the 50:50 samples, LFD images for PVDF:PE 20:80 10 min of mixing (Figure 11b) show ridges with increased size surrounding the PVDF droplets compared to the 1 min sample. The increase in the size of ridges around the PVDF droplets with increased mixing time matches the increased concentration of MWCNT agglomerates seen during TEM imaging (Figure 5), indicating that an increase in the concentration of MWCNTs building up at the PVDF/PE interface is causing the ridge formation at the edge of the droplets. These results confirm that during mixing, MWCNTs are diffusing through the PE phase to the interface and accumulating there.

### 3.3. DC Electrical Conductivity and EMI Shielding Effectiveness

**Figure 12:**
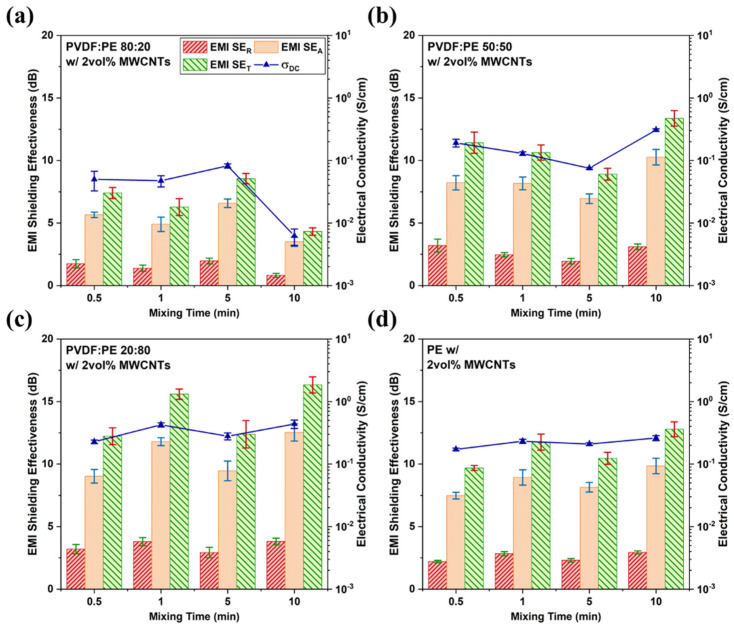
EMI SE values and DC conductivity readings for (**a**) 80:20, (**b**) 50:50, and (**c**) 20:80 samples with a thickness of 0.45 mm. A plot of (**d**) PE with 2 vol% MWCNTs is also included.

Figure 12 shows the effect of mixing time on the final observed DC electrical conductivity (σ_DC_) and EMI SE for each blend composition and for the pure PE composite, at a MWCNT concentration of 2 vol%. Previous works have shown that the EMI SE of a PBN is correlated to its conductivity, as both require a high dielectric loss [7]. The 80:20 samples (Figure 12a) show a reduction in σ_DC_ and EMI SE_T_ at a higher mixing time of 10 min, and the 80:20 samples also exhibit the lowest electrical properties for all mixing times. Since MWCNTs were pre-localized in the dispersed PE phase, their ability to form a conductive network within the blend is hindered, resulting in greatly diminished electrical properties. Despite this, a noticeable rise in σ_DC_ and EMI SE is seen at 5 min, which is indicative of some degree of MWCNT network formation. At 10 min of mixing, σ_DC_ and EMI SE drop significantly, well below the values seen at 30 s or 1 min. It is possible that the rise seen at 5 min of mixing is due to the formation of a MWCNT network with MWCNT-rich PE droplets acting as nodes for the network. The drop at 10 min may be due to a breakup of this transient MWCNT network, coupled with droplet coalescence resulting in an increase in the domain size of the PE phase, and reducing the number of nodes.

The 50:50 samples exhibit a modest and gradual drop in EMI SE and electrical conductivity up to the 5 min mark, and then rise significantly at 10 min of mixing. The LM images for the 50:50 samples (Figure 3e–h) show that there is co-continuity within the PE phase; however, the domain size of the PE phase at 1 min (Figure 3f) appears to be significantly smaller than at any other mixing time. This suggests that the initial co-continuous MWCNT network within PE is broken down by shear forces during mixing, resulting in reduced σ_DC_ and EMI SE. Past 5 min of mixing, MWCNTs begin concentrating significantly more along the PVDF/PE interface and by 10 min of mixing, new MWCNT networks have formed at the interface of the blend. The formation of these new MWCNT networks results in an increase in the observed σ_DC_ and EMI SE at 10 min of mixing.

The 20:80 samples (Figure 12c) exhibit similar behavior to the PE/MWCNT samples, which suggests a similar MWCNT network structure development occurs for these two systems. The 20:80 system is characterized by a rise in σ_DC_ and EMI SE between 30 s and 1 min of mixing, a drop at 5 min of mixing, and a final rise at 10 min. The initial rise between 30 s and 1 min of mixing is likely due to the breakdown of pre-exiting MWCNT network within the PE phase during initial mixing, as seen in LM images (Figure 3i,j). As these networks are broken down, more MWCNTs become available to be rearranged into networks within the PE phase. The drop at 5 min is likely due to accumulation of MWCNT at the interface, a phenomenon similar to that seen in the 50:50 sample at the same time. That is, the pre-existing MWCNT network breaks down as MWCNTs migrate toward the PVDF/PE interface, resulting in reduced values of σ_DC_ and EMI SE. After 10 min of mixing, MWCNT-coated PVDF droplets act as nodes for a new MWCNT network, resulting in increased values of σ_DC_ and EMI SE.

For all samples, the dominant mechanism of EMI attenuation was absorption, which is typical for MWCNT-based PBNs. The dominance of absorption within PBNs is because polymers inherently offer no shielding properties on their own, meaning that incident waves can penetrate the bulk of the sample. Once inside, the electric and magnetic fields of the electromagnetic wave interact with the electric and magnetic dipoles present within the embedded MWCNTs, resulting in attenuation through ohmic losses [42]. Absorption as the dominant mechanism of EMI attenuation represents a significant advantage for PBNs over traditional shielding materials such as metals that depend mainly on reflection to attenuate the incident radiation [8]. Via absorption, incident EMI is dissipated as heat within the PBN, rather than being reflected into the environment. Thus, PBN-based shielding materials reduce EM pollution (EMI smog) in the environment, contributing to human health and the safe operation of electronic devices [43].

### 3.4. Electrical Permittivity

**Figure 13:**
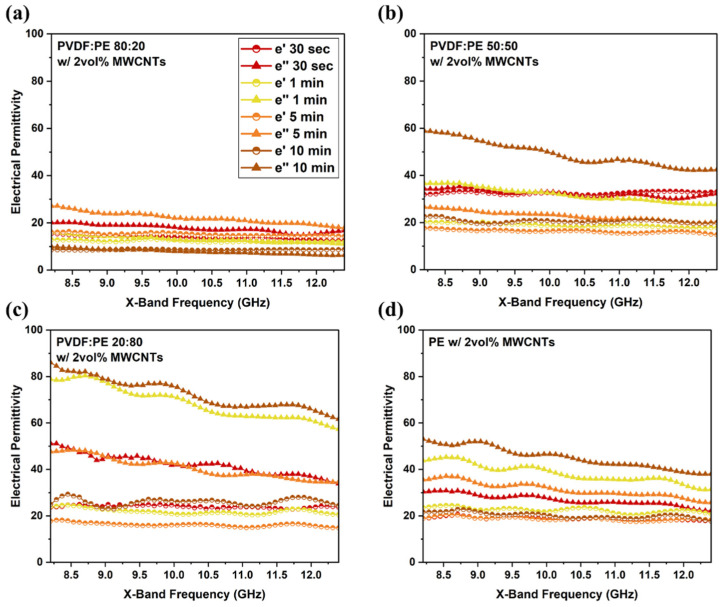
Electrical permittivity data across the X-band for (**a**) 80:20, (**b**) 50:50, and (**c**) 20:80 samples with a thickness of 0.45 mm. A plot of (**d**) PE with 2 vol% MWCNTs is also included.

The observed σ_DC_ and EMI SE of the materials prepared in this work can be studied in terms of their dielectric properties. Complex electrical permittivity, which is defined as ($\varepsilon^{*}=\varepsilon^{\prime }+{i\varepsilon }^{\prime \prime }$) or ($\varepsilon^{*}=\sqrt{{\left(\varepsilon {}^{\prime }\right)}^{2}+{\left(\varepsilon {}^{\prime \prime }\right)}^{2}}$), is comprised of the real permittivity ($\varepsilon^{\prime }$) and imaginary permittivity ($\varepsilon {}^{\prime \prime }$). In simple terms, the real permittivity of a material represents its ability to store electrical energy, and the imaginary permittivity represents its ability to dissipate energy (via attenuation). For PBN systems containing MWCNTs, $\varepsilon^{\prime }$ is the result of interfacial polarization between the polymer and MWCNTs, whereas $\varepsilon^{\prime \prime }$ is the result of attenuation due to dissipation through interconnected MWCNT networks [7]. Figure 13 shows the real and imaginary permittivity values for prepared PVDF:PE samples (Figure 13a–c), and PE composite (Figure 13d), all prepared at a MWCNT volume fraction of 2 vol%. The value of $\varepsilon {}^{\prime \prime }$ increases as the volume fraction of PE is increased, likely due to increased co-continuity of the PE phase throughout the mixing process, resulting in a greater amount of interconnected MWCNT networks. $\varepsilon^{\prime }$ appears to be far less impacted as the mixing time is increased. Since $\varepsilon^{\prime }$ depends on the interfacial area between MWCNTs and polymer, it changes less for the different systems [44,45,46]. Although we may expect large MWCNT agglomerates to reduce the interfacial area between MWCNTs and the polymer, since the polymer molecules are much smaller than MWCNTs, they can easily fit within the gaps between individual MWCNT within each agglomerate and thus, the interfacial area will change little between the different systems [47]. 

The 80:20 samples (Figure 13a) exhibit low $\varepsilon^{\prime }$ and $\varepsilon^{\prime \prime }$ values across the X-band, and a decreasing average value with increased mixing, matching the σ_DC_ and EMI SE data (Figure 12a). The high concentration of MWCNTs within the PE droplets results in the formation of numerous MWCNT agglomerates inside the droplets, which in turn leads to a slight reduction in interfacial polarization. Furthermore, because MWCNTs are concentrated within the PE phase, they cannot form interconnected networks spanning the sample, and consequently poor EMI shielding is observed. $\varepsilon^{\prime }$ and $\varepsilon^{\prime \prime }$ values for the 50:50 samples (Figure 13b) and 20:80 samples (Figure 13c) show the same trend as seen for the corresponding σ_DC_ and EMI SE data (Figure 12c,d). The increase in $\varepsilon {}^{\prime \prime }$ values in these systems is attributed to an increase in the amount of interconnectivity between MWCNTs within the system, because MWCNT-rich PE is co-continuous in these PBNs.

**Figure 14:**
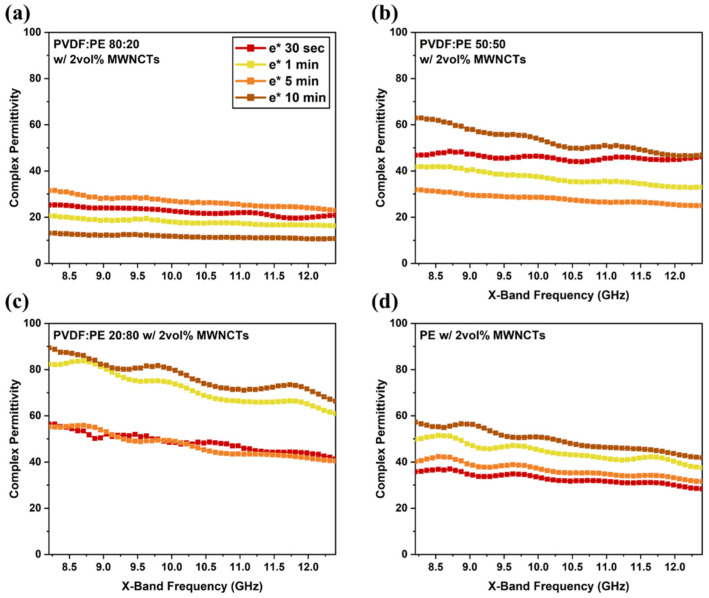
Complex permittivity data within the X-band for (**a**) 80:20, (**b**) 50:50, and (**c**) 20:80 samples with a thickness of 0.45 mm. A plot of (**d**) PE with 2 vol% MWCNTs is also included.

Since both $\varepsilon^{\prime }$ and $\varepsilon {}^{\prime \prime }$contribute to the ability of a given material to shield against EMI, studying the complex permittivity ($\varepsilon^{*}$) can provide additional insight into the overall shielding abilities of each material. Figure 14 shows the $\varepsilon^{*}$ data for all prepared PVDF:PE samples. The use of $\varepsilon^{*}$ highlights the similarities of $\varepsilon^{*}$ to EMI SE and σ_DC_ across all prepared samples. Based on Figure 12 and Figure 14, the 50:50 and 20:80 samples both achieve higher value of $\varepsilon^{*}$, EMI SE, and σ_DC_ after 10 min of mixing. This strongly suggests that at least 10 min of mixing is required to fully observe a MWCNT network along the PVDF/PE interface in these systems.

### 3.5. Rheological Properties

**Figure 15:**
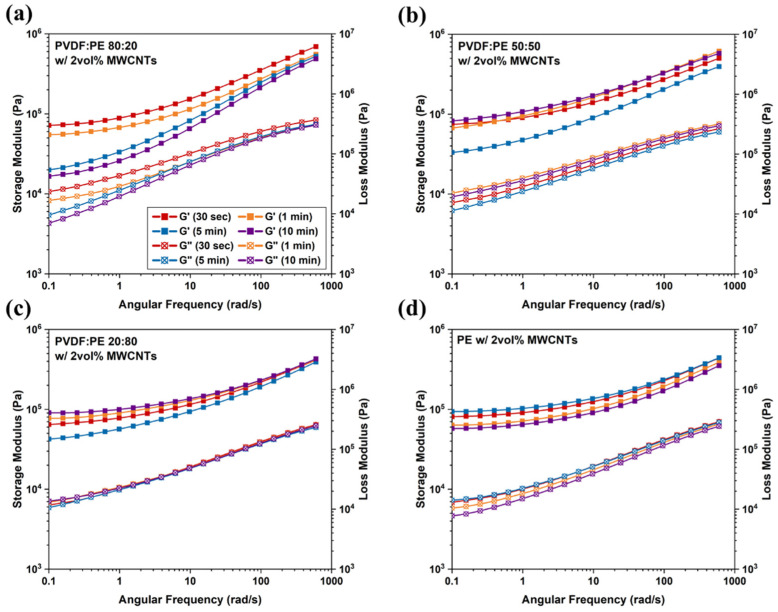
Frequency sweep results for (**a**) 80:20, (**b**) 50:50, and (**c**) 20:80 samples with a thickness of 0.45 mm. A plot of (**d**) PE with 2 vol% MWCNTs is also included.

Rheology is a powerful tool that has been widely used to correlate the macro-scale mechanical response of PBNs in their melt state to the nanostructures formed within the blend nanocomposite, namely nanofiller networks [48]. Oscillatory frequency sweeps at 0.5% strain in the LVE region were performed on the PVDF:PE blends, to measure the viscoelastic properties of the blends and detect MWCNT networks. Based on the results in Figure 15, for all mixing times and PVDF:PE sample compositions, the responses are predominantly elastic. However, the solid-like behavior of the samples is more pronounced as the volume fraction of PE increases. Since MWCNTs are pre-localized within the PE phase, there are more MWCNTs dispersed in the continuous phase. At higher PE concentrations, less variation is seen in the viscoelastic moduli with increased mixing time. 

The 80:20 samples exhibit a reduction in G’ as the mixing time is increased (Figure 15a), likely because of deterioration of the MWCNT network due to shear with increased mixing time. This trend matches EMI SE and electrical conductivity (Figure 12a) data. There is an anomaly at 5 min where the PBN has a low G’ value despite the relatively high electrical properties. It may be that the MWCNT network is sufficient for electrical properties but connections between polymer chains and MWCNT have broken down with shearing. The downward trend in G’ and G” values observed for PVDF:PE 80:20 samples is consistent with the increasing concentration of MWCNTs at the interface seen via TEM. Both the 50:50 and 20:80 samples (Figure 15b,c) showed G’ and G” values that match closely with the corresponding electrical properties, which confirms that the pre-existing MWCNT network in the PE phase initially breaks down, and subsequently a new MWCNT network forms along the PVDF/PE interface.

**Figure 16:**
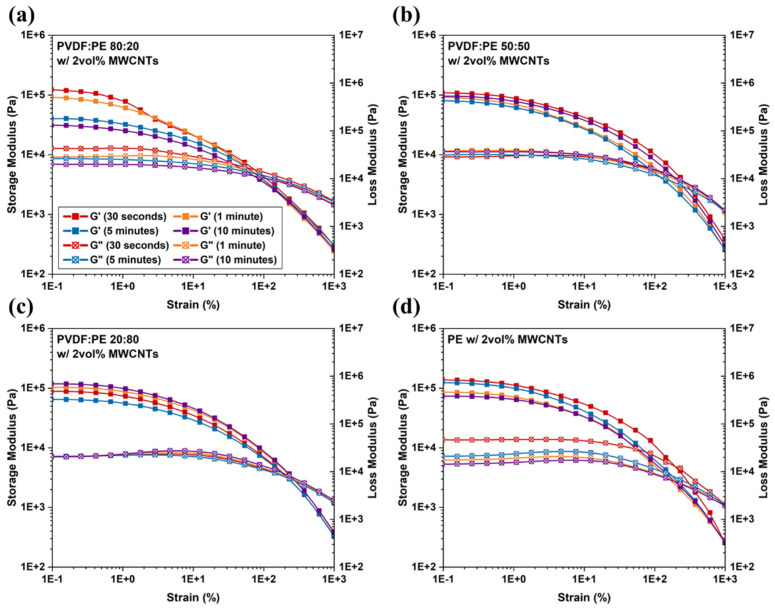
Strain sweep results for (**a**) 80:20, (**b**) 50:50, and (**c**) 20:80 samples with a thickness of 0.45 mm. A Plot of (**d**) PE with 2 vol% MWCNTs is also included.

Strain sweeps were conducted to confirm the LVE region and to link the inter-cycle nonlinear viscoelastic responses to MWCNT network structures contained within the PVDF:PE PBN samples. Figure 16 shows the relationship between the strain amplitude and the observed viscoelastic properties for PVDF:PE blends containing 2 vol% MWCNTs mixed for different times. The results show that at 0.5% strain, all the systems lie within the LVE region. The G’ values across all blend compositions are significantly higher than the corresponding G” values, meaning the systems have a greater elastic response than viscous response. As the volume fraction of PE is increased, the G’ values in the LVE region increase and the G” values decrease. When strain amplitude exceeds the critical value, G’ begins to decrease rapidly and these materials exhibit a crossover point (strain amplitude where G” becomes greater than G’) [49]. The decrease in G’ is typically associated with the destruction of the original nanofiller microstructure [50]. For both the 80:20 and 20:80 PVDF:PE samples (Figure 16a,c), increasing mixing time has little impact on the crossover point observed the strain sweep, suggesting a comparable rigidity of the network structure. However, in the case of the 50:50 blends compositions (Figure 16b), increasing mixing time influences the crossover strain, which suggests that a greater change in the MWCNT network structure occurs in the 50:50 blends during mixing. 

## 4. Discussion

### 4.1. MWCNT Migration Behavior

The variation of blend composition influences several parameters that affect the dispersion and final localization of MWCNTs within the prepared PVDF:PE samples. Because the overall volume fraction of MWCNTs is kept constant for all samples, and because MWCNTs are pre-localized in PE, varying the volume ratio of PVDF to PE directly impacts the concentration of MWCNTs within the PE phase at the onset of mixing. In the case of the PVDF:PE 80:20 samples, the volume concentration of MWCNTs within the PE phase is high, at around 9.3 vol%. This results in the MWCNT-rich PE phase resembling MWCNT agglomerates coated in PE. The agglomerates end up concentrating at the PVDF/PE interface during mixing, and individual MWCNTs penetrate the PVDF phase (Figure 4). Shearing forces during the mixing process likely allow individual MWCNTs to break away from their parent agglomerates, and these MWCNTs consequently migrate completely into the PVDF phase, since they are already straddling the interface. The high shear during mixing also causes scission of MWCNTs and some parts of MWCNTs in the agglomerates may break off and enter the PVDF phase. 

For the PVDF:PE 50:50 samples, the concentration of MWCNTs is significantly lower, approximately 3.9 vol%, within PE at the onset of mixing. The reduced local concentration of MWCNTs results in fewer MWCNT agglomerates present at the start of mixing and more individual MWCNTs. This coupled with the higher interfacial surface area shared between PVDF and PE due to their largely co-continuous morphology, results in a higher probability of individual MWCNTs encountering the PVDF/PE interface. Even at 30 s of mixing (Figure 5a,a1), many MWCNTs are seen poking into the PVDF phase. By 1 min of mixing (Figure 5b,b1), individual MWCNTs have already started to fully enter the PVDF phase, and MWCNT agglomerates can be seen concentrating near the PVDF/PE interface. This trend continues and by 10 min of mixing (Figure 5d,d1), dark MWCNT-rich rings can be seen concentrating around PVDF droplets. The reduction in the domain size of PVDF during this time frame suggests that the dark rings could be MWCNT-rich PVDF, rather than MWCNTs on the PE side of the interface.

The PVDF:PE 20:80 samples have the lowest concentration of MWCNTs in the PE phase at the onset of mixing (roughly 2.5 vol%) and thus, have the lowest amount of MWCNT agglomerates. Despite the relatively low interfacial surface area available between the PVDF droplets and the PE matrix within the 20:80 samples, the large amount of individual MWCNTs (as opposed to MWCNT agglomerates) within the PE phase aid in the effective migration of MWCNTs across the PVDF/PE interface into the PVDF phase. Although few MWCNTs can be seen straddling the PVDF/PE interface at 30 s (Figure 6a,a1), we can see much more at 1 min (Figure 6b,b1), and at 5 min of mixing (Figure 6c,c1), dark rings of MWCNT-saturated PE can be seen surrounding all the PVDF domains. At 10 min, MWCNTs have invaded some PVDF droplets. The larger fraction of individual MWCNTs reduces the amount of nanofiller congestion at the PVDF:PE interface. 

The commonality of the three PVDF:PE blends is the difference in the migration tendencies of MWCNT agglomerates and individual MWCNTs. Analysis of the TEM micrographs for all samples showed no MWCNT agglomerates present within the PVDF phase, across an area of 9 µm^2^ for each micrograph. In contrast, 10–20 individual MWCNTs are typically seen within the same micrograph area. This suggests significantly different migration dynamics for MWCNT agglomerates versus individual MWCNTs. MWCNT agglomerates are still drawn to the PVDF/PE interface and are seen concentrating at the interface during mixing (see Figure 4, Figure 5 and Figure 6), but they cannot penetrate the interface. Since both individual MWCNTs and MWCNT agglomerates are drawn toward the polymer blend interface, it is likely the discrepancies in their geometry that can explain their final localization within the blend. 

### 4.2. Modified “Slim-Fast Mechanism”

**Figure 17:**
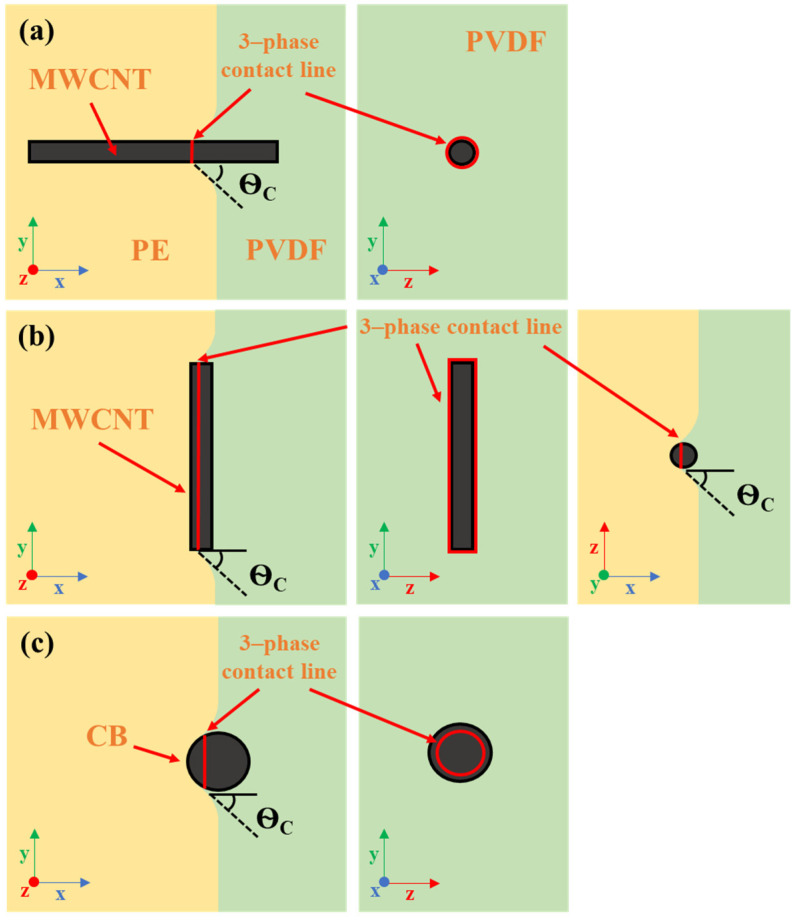
Three-phase contact line in a PVDF/PE blend for (**a**) a MWCNT perpendicular to the interface, (**b**) a MWCNT parallel to the interface, and (**c**) a CB particle. Adapted from [34]. Copyright 2022 American Chemical Society.

The underlying mechanisms of MWCNT migration across the PVDF/PE interface can be explained via Göldel et al.’s Slim-Fast Mechanism (SFM). According to SFM, low AR nanoparticles are less able to penetrate polymer blend interfaces than high AR nanoparticles [34]. The basis of SFM is built on a previous work by Krasovitski and Marmur [51], that used a penetration index to quantify the tendency of a spheroidal particle to transfer across a liquid/liquid interface. It was found that as the aspect ratio of a particle was increased, the relation between the penetration index and the contact angle between the particle and the destination liquid approached a stepwise function. This suggests that high AR particles that approach an interface perpendicular to the plane can easily penetrate the interface, if the interfacial energy of the destination phase is not excessively high (i.e., contact angle, $\theta_{C}\geq 90{}^{\circ}$). Furthermore, it was also shown that positive line tension had a significant impact on the penetration index for nanoscale particles, whereas negative line tension had a much smaller effect. Line tension is defined as the tension that acts over the three phase contact line within a ternary system [52]. In the case of a PVDF/PE system containing nanofillers, the three-phase contact line is the interfacial perimeter of contact between PVDF, PE and the nanofiller. Figure 17 outlines the three-phase contact line for (1) a MWCNT approaching perpendicular to the interface, (2) a MWCNT approaching parallel to the interface, and (3) a CB particle approaching the interface.

A three-phase system containing two polymer phases (A and B), and a single nanoparticle phase, can be described in terms of its free energy [51]. By differentiating with respect to the x-axis (migration path), and setting the Gibbs free energy to a minimum, $\frac{\mathrm{dG}}{\mathrm{dx}}=0$, the equilibrium state of the particle can be found:

(2) $$\frac{{\mathrm{dA}}_{\mathrm{ab}}}{\mathrm{dx}}=\frac{{\mathrm{dA}}_{\mathrm{pb}}}{\mathrm{dx}}\cos\left(\theta_{C}\right)-\tau \frac{\mathrm{dL}}{\mathrm{dx}}$$

where $\frac{{\mathrm{dA}}_{\mathrm{ab}}}{\mathrm{dx}}$ corresponds to the free energy contribution of the polymer A/polymer B interface, $\frac{{\mathrm{dA}}_{\mathrm{pb}}}{\mathrm{dx}}\cos\left(\theta_{C}\right)$ corresponds the free energy contribution of the polymer B/particle interface, and $\tau \frac{\mathrm{dL}}{\mathrm{dx}}$ corresponds to free energy contribution due to line tension acting along L. For more details on the derivation of Equation (2), and the definition of the accompanying variables, please refer to the Supplementary Materials.

If Equation (2) cannot be satisfied, then the filler will either fully migrate into polymer B or remain on the polymer A side. In the case of a nanoparticle pre-localized in the PE phase of a PVDF/PE blend, it will begin to migrate toward the interface with PVDF. Once the nanoparticle is close to the interface, PVDF will begin to wet the nanoparticle. This wetting of the particle will create instability at the interface, which is caused by the increased surface area between PVDF and PE caused by the contact angle between the nanofiller and PVDF. The extent of the particle’s penetration will depend on several factors, including the geometry of the particle and particle trajectory (e.g., particle shape, angle of approach, etc.), the line tension and the contact angle between the particle and PVDF.

**Figure 18:**
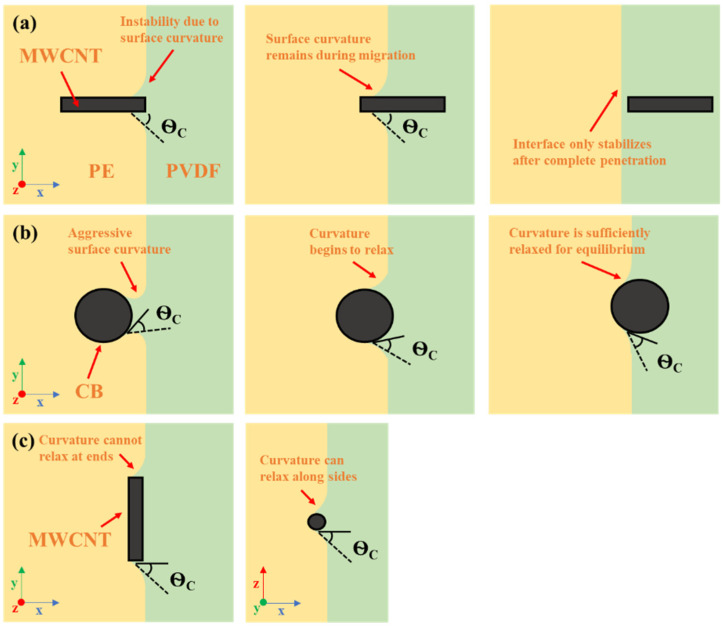
Migration progression for (**a**) a MWCNT approaching perpendicular to the PVDF/PE interface, (**b**) a CB particle approaching the interface, and (**c**) a MWCNT approaching parallel to the interface. Adapted from [34]. Copyright 2022 American Chemical Society.

Figure 18 shows the migration progression for different nanoparticles based on the behavior proposed by SFM [34]. In the case of a perpendicular MWCNT (Figure 18a) migrating along the x-axis, the curvature of the MWCNT exists only in the y–z plane, which is perpendicular to the path of migration. Because of this, the PVDF interface cannot relax once it begins wetting the MWCNT. In terms of the free energy balance, $\frac{{\mathrm{dA}}_{\mathrm{ab}}}{\mathrm{dx}},\;\frac{\mathrm{dL}}{\mathrm{dx}}=0$, since the PVDF surface area, and the wetting perimeter remain constant. $\frac{{\mathrm{dA}}_{\mathrm{pb}}}{\mathrm{dx}}>0$, and thus Equation (2) cannot be satisfied. Thus, the only way to stabilize the interface is to continue wetting the MWCNT and pulling it through, until it fully enters PVDF. For a CB particle (Figure 18b) migrating along the x-axis, the curvature of the particle exists in all planes. Because of this, the PVDF interface continuously relaxes as it begins to wet and pull in the CB particle. In terms of the free energy balance, $\frac{{\mathrm{dA}}_{\mathrm{ab}}}{\mathrm{dx}},\;\frac{\mathrm{dL}}{\mathrm{dx}}\neq 0$, since the curvature is constantly decreasing, and the wetting perimeter is constantly increasing. Consequently, once the CB has penetrated far enough into PVDF, the PVDF interface will become sufficiently relaxed for equilibrium to be achieved. Finally, in the case of a parallel MWCNT (Figure 18c) migrating along the x-axis, the curvature of the MWCNT exists in the x–z plane which means that the PVDF interface can relax, as seen in the case of the CB particle. Similarly, to the CB particle, $\frac{{\mathrm{dA}}_{\mathrm{ab}}}{\mathrm{dx}},\;\frac{\mathrm{dL}}{\mathrm{dx}}\neq 0$. Although the curvature does not relax in the x–y plane, because the domain of the interface that relaxes acts along the length of the MWCNT (most of its surface area), the particle is still able to achieve equilibrium at the interface. Another factor that must be considered is line tension. In the case of positive line tension, it is difficult to induce curvature along a large perimeter, since it results in a larger increase in the interfacial area between the two immiscible polymer phases. CB particles and perpendicular MWCNTs create small wetting perimeters when wetted by PVDF, meaning that the impact of line tension is less significant. Parallel MWCNTs present a far more significant wetting perimeter. This high wetting perimeter results in a greater line tension occurring at the three-phase contact line, which impedes the continued migration of the MWCNT in the PVDF phase.

**Figure 19:**
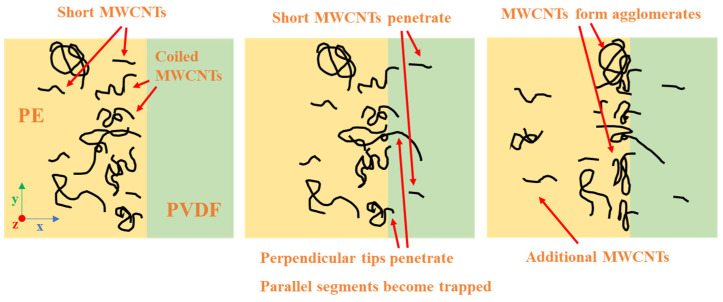
Proposed large-scale migration behavior of MWCNTs in PVDF/PE blend system. Short MWCNTs near interface successfully enter PVDF, whilst large coiled MWCNTs and MWCNT agglomerates become trapped.

The three examples presented in Figure 18 (CB, perpendicular MWCNTs, and parallel MWCNTs) all represent ideal nanoparticles. For MWCNTs, the reality is far different. The extremely high AR of MWCNTs causes them to coil on themselves, and intermolecular forces often cause MWCNTs to form agglomerates during mixing. These complex arrangements of MWCNTs can be thought of as a combination of parallel and perpendicular segments. Any peripheral MWCNT sections that protrude from a given coiled MWCNT or MWCNT agglomerate are more likely to be wetted by PVDF and cross the interface, whilst the parallel segments will become trapped at the interface. Figure 19 outlines the proposed behavior of MWCNTs within the PVDF/PE blends as they approach the interface. Short MWCNTs near the interface are more likely to be wetted by PVDF and pulled into the PVDF phase. Coiled MWCNTs are far more likely to become trapped since they will contain segments parallel to the PVDF/PE interface. These trapped MWCNTs can act as barriers for other incoming MWCNTs, forming networks along the PVDF/PE interface. 

Based on this mechanism, coiled MWCNTs and MWCNT agglomerates are desirable for achieving interfacial localization. The size of MWCNT agglomerates can be tuned via controlling the mixing conditions during masterbatch preparation. Although MWCNT agglomerates are the consequence of inefficiencies in the dispersion process during melt mixing, their presence could be of great use for encouraging the interfacial localization of MWCNTs within binary polymer blends. Tuning the size of MWCNT agglomerates via optimized processing strategies could pave the way to develop PBNs with small MWCNT agglomerates localized at the polymer/polymer interface.

## 5. Conclusions

In this work, the effects of blend composition and mixing time on the phase migration characteristics of MWCNTs from a polyethylene (PE) phase to a poly(vinylidene fluoride) (PVDF) phase were studied. The role of the MWCNT localization in morphology development, electrical properties, and rheological properties were investigated. A two-step mixing procedure was used to pre-localize MWCNTs in the less thermodynamically favored PE phase and to allow MWCNTs to migrate from the PE phase into the PVDF phase. PVDF:PE blends containing 2 vol% MWCNTs were prepared and characterized using TEM, which shows that MWCNTs migrate toward the PVDF phase as the mixing time increases. Individual MWCNTs fully migrated into the PVDF phase, while MWCNT agglomerates remained trapped at the interface. 

The PVDF:PE 50:50 and 20:80 samples showed high total EMI SE (13.4 dB and 16.3 dB, respectively) and electrical conductivity (of 3.1 × 10^−1^ and 4.4 × 10^−1^ S/cm, respectively) values after 10 min of mixing. This was due to the high amount of co-continuity of the PE phase in these blends. When the MWCNTs concentrated at the PVDF/PE interface during mixing, they began to percolate (i.e., forming a network), which resulted in a significant increase in the observed conductivity and EMI SE at 10 min of mixing, when compared to the pure PE nanocomposite (12.8 dB and 2.6 × 10^−1^ S/cm). Ultimately, TEM images showed that MWCNTs migrate toward the PVDF/PE interface, suggesting that thermodynamic predictions of MWCNTs’ preference for PVDF were confirmed. Both individual MWCNTs and MWCNT agglomerates were observed to migrate toward the PVDF/PE interface. A modified “Slim-Fast-Mechanism” was proposed to explain the differences in migration behavior of individual MWCNTs and MWCNT agglomerate. According to this theory, straight, individual MWCNTs that approach perpendicular to the interface have a higher likelihood of penetrating fully into the PVDF phase, while coiled MWCNTs and MWCNT agglomerates tend to be trapped and remain at the interface. Once at the interface, it was proposed that trapped MWCNTs acted as barriers preventing migrating MWCNTs from penetrating the PVDF/PE interface. The proposed mechanism was in accordance with several prior works and suggested the feasibility of using MWCNT agglomerates to achieve interfacial localization of MWCNTs within immiscible polymer blends.

## Figures and Tables

**Table 1:** Surface energies and wettability MWCNTs within PVDF and PE at 200 °C.

Parameter	Geometric Mean ^1^	Harmonic Mean ^2^
σ_PVDF/PE_ (mJ/m^2^)	7.00	7.00
σ_PVDF/MWCNT_ (mJ/m^2^)	11.74	12.26
σ_MWCNT/PE_ (mJ/m^2^)	27.18	27.46
Wettability	−2.21	−2.17

^1,2^ Wettability parameters were calculated using data collected from Wu [40] and Owens [41].

## Data Availability

The data presented in this are available upon request from the corresponding author.

## Supplementary Materials

The following are available online. Table S1: Surface energy values for each component within the PVDF/PE system at 200 °C [40,41], Figure S1: Frequency sweep data and strain sweep data for pure PVDF and PE prepared at a thickness of 0.45 mm, Figure S2: EMI SE and DC conductivity data for PVDF nanocomposites containing 2 vol% MWCNTs prepared with a thickness of 0.45 mm, Figure S3: Permittivity data for PVDF nanocomposites containing 2 vol% MWCNTs prepared with a thickness of 0.45 mm, including real and imaginary permittivity, and complex permittivity, Figure S4: Frequency sweep data and strain sweep data for PVDF nanocomposites containing 2 vol% MWCNTs prepared with a thickness of 0.45 mm. References [7,51] are cited in the supplementary materials.

## Sample Availability

Samples of the composites prepared in this work are available from the corresponding author.
