# Supplementary material for: The Role of Phase Migration of Carbon Nanotubes in Melt-Mixed PVDF/PE Polymer Blends for High Conductivity and EMI Shielding Applications
Source: Molecules. 2022 Jan 29;27(3):933. doi: 10.3390/molecules27030933 (PMC8839367; doi:10.3390/molecules27030933)
Supplement: Supplementary file 1 [file molecules-27-00933-s001.zip › molecules-1547560-supplementary/MDPI Molecules Paper - Nomenclature.pdf]

Nomenclature

EM – electromagnetic radiation

EMI – electromagnetic interference

CNT – carbon nanotube

MWCNT – Multiwalled carbon nanotubes

PNC – polymer nanocomposite

AR – aspect ratio

PBN – polymer blend nanocomposite

CB – carbon black

$\omega_{A\ to\ B}$ - wettability of nanofiller within a binary blend of polymer A and B (unitless)

$\sigma_{A/B}$ - surface energy between component A and B (mJ/m^2)

PS – polystyrene

ABS – acrylonitrile butadiene styrene

PA12 – polyamide 12

PE – polyethylene

PLA – polylactic acid

PCL – polycaprolactone

PVDF – poly(vinylidene difluoride)

LM – light microscope

TEM – transmission electron microscope

MB – masterbatch

APAM – Alberta Polymer Asymmetric Mini-mixer

BSE – backscattered electron

SEM – scanning electron microscope

LFD – large field detector

EMI SE – electromagnetic interference shielding effectiveness (dB)

EMI SE_A – EMI SE due to absorption (dB)

EMI SE_R – EMI SE due to reflection (dB)

EMI SE_{MR} – EMI SE due to multiple reflections (dB)

EMI SE_T – Total EMI SE (dB)

LVE – linear viscoelastic region

σ_{DC} - DC electrical conductivity (S/cm)

ε' - real permittivity (unitless, relative to permittivity of free space)

ε'' - imaginary permittivity (unitless, relative to permittivity of free space)

ε^* - complex permittivity (unitless, relative to permittivity of free space)

G' – storage modulus (Pa)

G" – loss modulus (Pa)

SFM – "Slim-Fast Mechanism"

θ_C – contact or wetting angle (°)

$\frac{dG}{dx}$ – change in Gibbs free energy with respect to migration path (J/m)

$\frac{dA_{ab}}{dx}$ – change in polymer A/polymer B interfacial area with respect to migration path (m^2/m)

$\frac{dA_{pb}}{dx}$ – change in polymer A/polymer B interfacial area with respect to migration path (m^2/m)

τ – 3-phase contact line between polymer A, polymer B and nanofiller (m)

$\frac{dL}{dx}$ – change in penetration length of nanofiller with respect to migration path (m/m)
