# Supplementary material for: The Role of Phase Migration of Carbon Nanotubes in Melt-Mixed PVDF/PE Polymer Blends for High Conductivity and EMI Shielding Applications
Source: Molecules. 2022 Jan 29;27(3):933. doi: 10.3390/molecules27030933 (PMC8839367; doi:10.3390/molecules27030933)
Supplement: Supplementary file 1 [file molecules-27-00933-s001.zip › molecules-1547560-supplementary/MDPI Molecules Paper - Supplementary Materials.pdf]

Supplementary Materials

1. Details on predicting thermodynamic affinity

The geometric and harmonic means derived by Wu [1] can be used as follows:

$$\sigma_{12,\text{harmonic}} = \sigma_1 + \sigma_2 - 4\left[\frac{\sigma_1^d \sigma_2^d}{\sigma_1^d + \sigma_2^d} + \frac{\sigma_1^p \sigma_2^p}{\sigma_1^p + \sigma_2^p}\right] \text{ (eq. S1)}$$

$$\sigma_{12,\text{geometric}} = \sigma_1 + \sigma_2 - 4\left[\frac{\sigma_1^d \sigma_2^d}{\sigma_1^d + \sigma_2^d} + \frac{\sigma_1^p \sigma_2^p}{\sigma_1^p + \sigma_2^p}\right] \text{ (eq. S2)}$$

Where σ_i is the total surface energy of component i, σ_i^d is the dispersive surface free energy of component i, and σ_i^p represents the polar surface free energy of component i. The individual surface energies for each component are summarized in Table .

Table S1. Surface energy values for each component within the PVDF/PE system at 200°C [1,2] .

**Component**	**Total Surface Free Energy (mJ/m^2)**	**Dispersive Surface Free Energy (mJ/m^2)**	**Polar Surface Free Energy (mJ/m^2)**
PVDF	30.3	23.3	7.00
PE	35.3	35.3	0.00
MWCNTs	45.3	18.4	26.9

2. EMI Scattering Parameter Calculations

Scattering parameters measured by the network analyzer were used to determine the EMI SE values for the prepared samples. $EMI\ SE_R$ and $EMI\ SE_A$ were calculated from scattering parameters as follows [3]:

$$R = \left|\frac{P_R}{P_I}\right| = |S_{11}|^2 = |S_{22}|^2 \text{ (eq. S3)}$$

$$T = \left|\frac{P_T}{P_I}\right| = |S_{12}|^2 = |S_{21}|^2 \text{ (eq. S4)}$$

$$\text{EMI SE}_R = 10 \cdot \log\left(\frac{1}{1-R}\right) \text{ (eq. S5)}$$

$$\text{EMI SE}_A = 10 \cdot \log\left(\frac{1-R}{T}\right) \text{ (eq. S6)}$$

Where R is the reflectance, T is the transmittance, P_R is the power of the reflected wave, P_I is the power of the incident wave and P_T is the power of the transmitted wave. Additionally, S_{11}, S_{12}, S_{21} and S_{22} are the forward reflection coefficient, forward transmission coefficient, reverse transmission coefficient and the reverse reflection coefficient, respectively.

3. Frequency and Strain sweep data for Pure PVDF and PE

Frequency and strain sweeps were performed on PVDF and PE to properly grasp their relative viscosities, and the results are plotted in Figures A2 and A3.

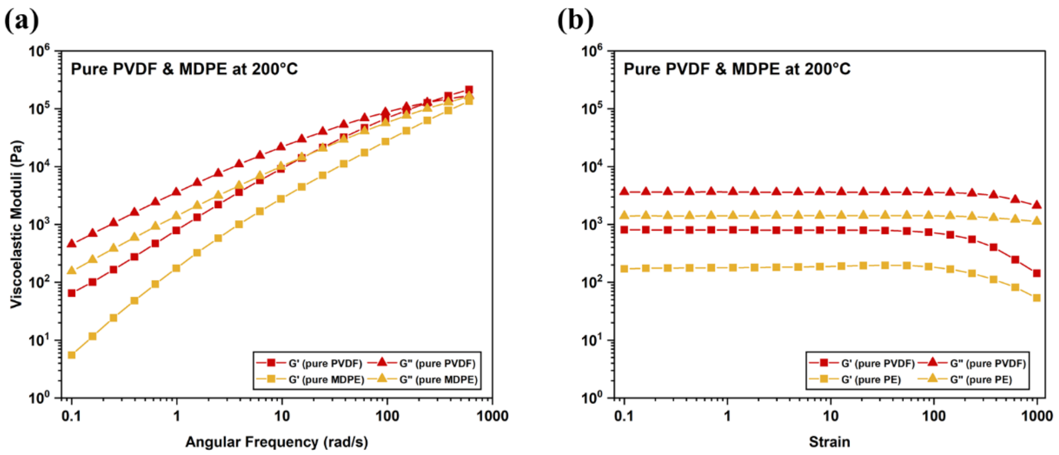

Figure S1. Frequency sweep data (**a**) and strain sweep (**b**) data for pure PVDF and PE prepared at a thickness of 0.45 mm.

4. PVDF Nanocomposite with 2vol% MWCNTs Data

In addition to preparing blends of PVDF:PE and pure PE nanocomposite, samples of PVDF nanocomposite were prepared and characterized to serve as an additional baseline for performance. The electrical and rheological results are plotted below.

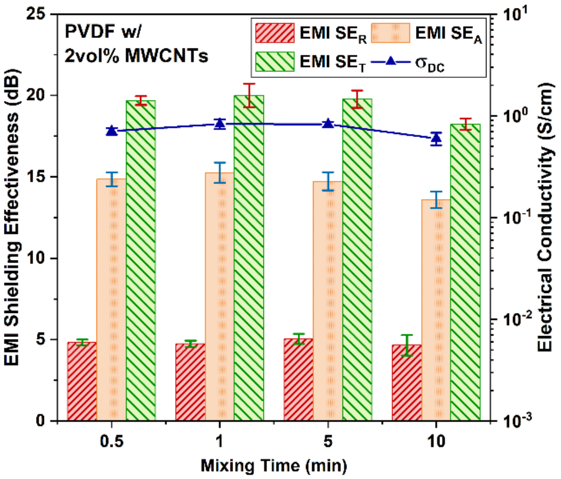

Figure S2. EMI SE and DC conductivity data for PVDF nanocomposites containing 2 vol% MWCNTs prepared with a thickness of 0.45 mm.

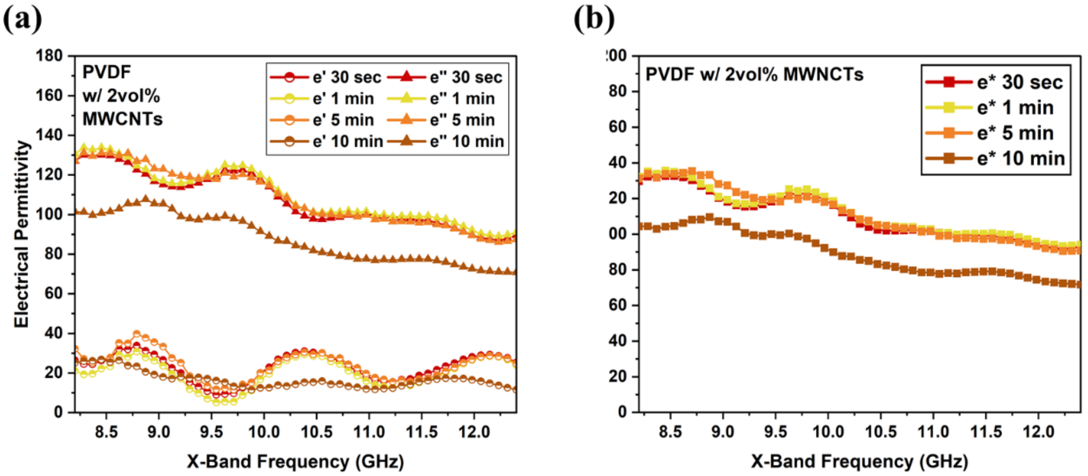

Figure S3. Permittivity data for PVDF nanocomposites containing 2 vol% MWCNTs prepared with a thickness of 0.45 mm, including real and imaginary permittivity (**a**), and complex permittivity (**b**).

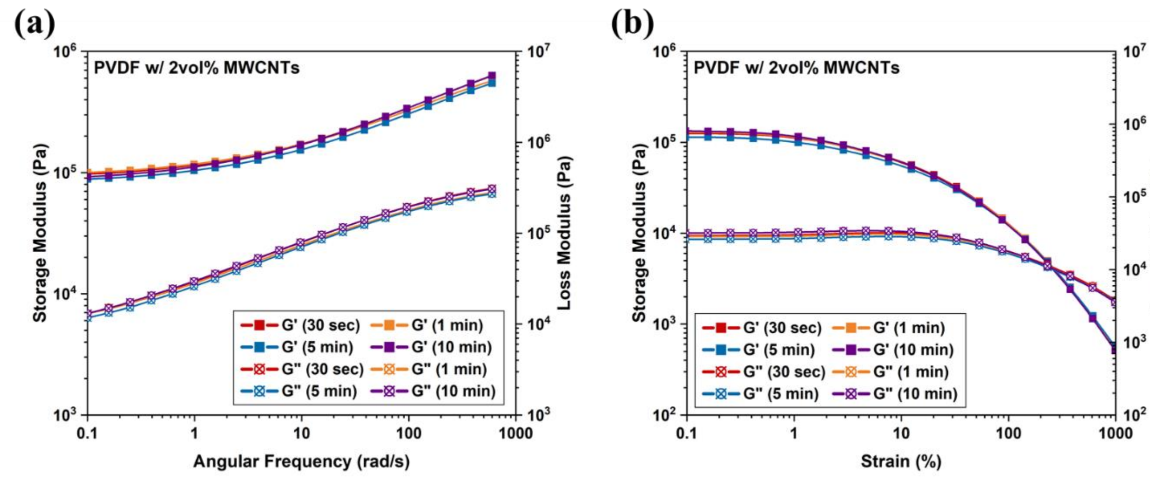

Figure S4. Frequency sweep data (**a**) and strain sweep data (**b**) for PVDF nanocomposites containing 2 vol% MWCNTs prepared with a thickness of 0.45 mm.

5. Derivation of Nanoparticle Equilibrium State

The energy balance for a three-phase system containing two polymer phases (A and B), and a single nanoparticle phase can be described as follows [4]:

$$\mathrm{G} = \sigma_{ab}\left(-A_{pb}\cos(\theta_C) + A_{ab} + \tau L\right) \text{ (eq. S7)}$$

Where G is the Gibbs free energy, σ_{ab} is the polymer A/polymer B interfacial tension, A_{pb} is the nanoparticle/polymer B surface area, A_{ab} is the polymer A/polymer B surface area, and τ is the line tension

acting along the 3-phase contact line, L. By differentiating with respect to the x-axis (migration path), and setting the Gibbs free energy to a minimum, $\frac{dG}{dx} = 0$, the equilibrium state of the particle can be found:

$$\frac{dG}{dx} = \sigma_{ab}\left(-\frac{dA_{pb}}{dx}\cos(\theta_C) + \frac{dA_{ab}}{dx} + \tau\frac{dL}{dx}\right) = 0 \quad (eq.\, S8)$$

$$\frac{dA_{ab}}{dx} = \frac{dA_{pb}}{dx}\cos(\theta_C) - \tau\frac{dL}{dx} \quad (eq.\, S9)$$

Where $\frac{dA_{ab}}{dx}$ corresponds to the free energy contribution of the polymer A/polymer B interface, $\frac{dA_{pb}}{dx}\cos(\theta_C)$ corresponds the free energy contribution of the polymer B/particle interface, and $\tau\frac{dL}{dx}$ corresponds to free energy contribution due to line tension acting along L.

6. References

1. S. Wu, *Polymer Interface and Adhesion*. New York: Marcel Dekker Inc., 1982.
2. D. K. Owens and R. C. Wendt, "Estimation of the surface free energy of polymers," *Journal of Applied Polymer Science*, vol. 13, no. 8, pp. 1741-1747, 1969.
3. S. P. Pawar, S. Biswas, G. P. Kar, and S. Bose, "High frequency millimetre wave absorbers derived from polymeric nanocomposites," *Polymer*, vol. 84, no. 10, pp. 398-415, 2015.
4. B. Krasovitski and A. Marmur, "Particle adhesion to drops," *The Journal of Adhesion*, vol. 81, no. 7-8, pp. 869-880, 2005.
