# Supplementary material for: The Role of Phase Migration of Carbon Nanotubes in Melt-Mixed PVDF/PE Polymer Blends for High Conductivity and EMI Shielding Applications
Source: Molecules. 2022 Jan 29;27(3):933. doi: 10.3390/molecules27030933 (PMC8839367; doi:10.3390/molecules27030933)
Supplement: Supplementary file 1 [file molecules-27-00933-s001.zip › molecules-1547560-supplementary/MDPI_Molecules_Paper_ToC.pdf]

Table of Contents

1. Introduction 1
2. Materials and Methods 3
2.1. Materials and Sample Preparation 3
2.2. Sample Characterization 4
3. Results 5
3.1. Predicting Thermodynamic Affinity 5
3.2. Imaging Results 5
3.3. DC Electrical Conductivity and EMI Shielding Effectiveness 12
3.4. Electrical Permittivity 13
3.5. Rheological Properties 15
4. Discussion 17
4.1. MWCNT Migration Behavior 17
5. Conclusions 21
References 22
